# Navigating through human‐induced biases in radiation oncology: Implementing advanced data strategies for precision in ML‐based auto‐contouring

**DOI:** 10.1002/acm2.14227

**Published:** 2023-11-27

**Authors:** Alhassan Mohammed Baidoo, Ruth Beulah Awotwe, Stephanie Brako Boateng, Samuel Nii Adu Tagoe, Philip Odonkor, Anna Mamoud

**Affiliations:** ^1^ Department of Medical Physics Sweden Ghana Medical Centre Accra Ghana; ^2^ Department of Radiography University of Ghana Accra Ghana; ^3^ Department of Medical Physics National Centre for Radiotherapy and Nuclear Medicine Korle Bu Teaching Hospital Accra Ghana

In recent decades, radiotherapy (RT) has emerged as an instrumental modality in oncological management, being utilized in an estimated 50% of all cancer cases. The technological advancements in radiotherapy delivery methods have enabled precise dose conformity to tumor volumes, while concurrently minimizing radiation exposure to the surrounding healthy tissue. Consequently, accurate delineation of the target volume including tumor‐involving nodal regions and organs at risks (OARs) has become imperative to improve tumor control and safeguard healthy tissue. The workflow in RT begins with contouring of CT images followed by treatment planning. Contouring is performed and reviewed manually slice by slice, by a radiation oncologist, a process that is inherently subjective, labor intensive and time consuming. Moreover, the quality and time used in contouring is based solely on the experience of the user.

Anatomical atlases and standard guidelines have partially addressed the challenge of contouring in radiotherapy (RT), yet realizing the full benefits of advancements in treatment planning in RT, ensuring accurate segmentation, enhancing planning efficiency and mitigating impacts on survival and quality of life.

Machine learning, a subset of artificial intelligence, has gained significant attention for its potential to revolutionize various industries, including healthcare. In radiotherapy, machine learning holds the promise of automating critical tasks, such as target volume delineation and treatment planning. Deep learning in machine learning, powered by neural networks, have the capacity to analyze medical images, recognize relevant structures, and create highly tailored treatment plans with unprecedented efficiency. However, as with any technology, challenges persist, and in the case of radiotherapy, a major concern lies in the quality and accuracy of the training data.

The cornerstone of machine learning in radiotherapy lies in the training data used to develop and fine‐tune the models. This data encompasses a range of medical images, including CT scans, MRIs, and PET scans, as well as crucial human‐drawn contours. The imaging dataset serve as a canvas on which contours are manually drawn by clinicians to delineate the target volumes and organs at risk. However, the human factor introduces a challenge—the potential for erroneous contours which can ultimately affect the accuracy of the trained model. Clinician delineated contours can have multiple error sources, including but not limited to:

1. Inter‐observer variability: Different clinicians, even when using the same guidelines, may interpret medical images differently. Variations in contour placement can occur due to differences in clinical judgment, expertise, and subjective interpretation of image features. These inconsistencies can introduce significant variability in treatment plans and ultimately affect patient outcomes.
2. Clinician experience: The level of experience and training of clinicians can influence the accuracy of contouring. Novice clinicians may be more prone to errors, while experienced practitioners may exhibit a greater level of precision. However, even experienced clinicians are not immune to occasional errors.
3. Annotation mistakes: Contouring involves manually tracing regions on medical images, which may result in inadvertent mistakes. These mistakes can range from simple slip‐ups in drawing lines to more complex issues, such as contouring the wrong structure entirely. Such errors can lead to suboptimal treatment plans and potentially harmful consequences for patients.
4. Deadlines: The time constraints inherent in clinical practice can also contribute to contour errors. Clinicians may be under pressure to complete contouring quickly, increasing the likelihood of errors, particularly in cases where complex structures or intricate details are involved.
5. Inherent imaging challenges: The quality and characteristics of the medical images themselves can pose challenges. Factors like image artifacts, noise, and indistinct boundaries can make contouring more error‐prone.

The conventional reliance on clinician‐drawn contours for training deep learning models in radiotherapy is associated with significant challenges and potential errors. The characteristics and quality of the training data have a profound influence on the performance of deep learning models in radiotherapy. Models trained on data containing contour mistakes may inadvertently inherit these errors, affecting the accuracy and reliability of treatment plans generated. Thus, while machine learning in radiotherapy holds great promise, it is essential to address and mitigate the potential errors associated with the human‐generated training data, ultimately leading to more accurate and reliable auto‐segmentation and treatment planning systems.

Moreover, there currently lacks formal education, training, and job planning for clinical staff to facilitate the implementation of automatic segmentation and ongoing assessment. Literature scarcely reports the perspectives of oncologists, RTTs, and additional clinical staff concerning these elements. Several surveys are in progress to aggregate views, perceptions, and the present application of, and barriers to, employing ML auto‐contouring in RT, aiming to utilize these insights for the development and deployment of auto‐contouring in clinical settings. Nevertheless, a comprehensive perspective seeks to evaluate the holistic use and impact of artificial intelligence (AI) in radiation oncology, beyond merely auto‐contouring.

In the ongoing battle against cancer, the integration of deep learning technologies and the mitigation of human induced contour errors represent significant strides towards better patient care and improved treatment outcomes. The potential of machine learning in radiotherapy lies in its ability to complement human expertise and not merely replace it. By embracing alternative training strategies, the field can overcome the pitfalls of contour‐based training and propel radiotherapy to new heights of patient care and safety.

## AUTHOR CONTRIBUTIONS

Alhassan Mohammed Baidoo: Conception of research idea, research design, literature review, correction, and approval of final manuscript. Ruth Beulah Awotwe: Research conception, research design, and review of initial draft. Stephanie Brako Boateng: Conception of research idea, literature review, and writing of initial draft. Samuel Nii Adu Tagoe: Research design and manuscript review. Philip Odonkor: Review and correction of initial draft. Anna Mamoud: Review of final manuscript.

